# Empirical evaluation of human fetal fMRI preprocessing steps

**DOI:** 10.1162/netn_a_00254

**Published:** 2022-07-01

**Authors:** Lanxin Ji, Cassandra L. Hendrix, Moriah E. Thomason

**Affiliations:** Department of Child and Adolescent Psychiatry, New York University School of Medicine, New York, NY, USA; Department of Population Health, New York University School of Medicine, New York, NY, USA; Neuroscience Institute, New York University School of Medicine, New York, NY, USA

**Keywords:** Fetal fMRI, Preprocessing, Normalization, Denoising, Smoothing, Functional connectivity

## Abstract

Increased study and methodological innovation have led to growth in the field of fetal brain fMRI. An important gap yet to be addressed is optimization of fetal fMRI preprocessing. Rapid developmental changes, imaged within the maternal compartment using an abdominal coil, introduce novel constraints that challenge established methods used in adult fMRI. This study evaluates the impact of (1) normalization to a group mean-age template versus normalization to an age-matched template; (2) independent components analysis (ICA) denoising at two criterion thresholds; and (3) smoothing using three kernel sizes. Data were collected from 121 fetuses (25–39 weeks, 43.8% female). Results indicate that the mean age template is superior in older fetuses, but less optimal in younger fetuses. ICA denoising at a more stringent threshold is superior to less stringent denoising. A larger smoothing kernel can enhance cross-hemisphere functional connectivity. Overall, this study provides improved understanding of the impact of specific steps on fetal image quality. Findings can be used to inform a common set of best practices for fetal fMRI preprocessing.

## INTRODUCTION

Understanding of human brain development has grown rapidly with the introduction of fetal resting-state functional connectivity (RSFC) (van den Heuvel & Thomason, 2016). In 2011, Veronica Schöpf and colleagues published the first fetal RSFC study (Schöpf et al., 2011), demonstrating that it was possible to noninvasively image whole-brain functional systems prior to birth by using MRI. Before this time, very few studies had measured fetal brain activity (Anderson & Thomason, 2013). Indeed, most of what was known about prenatal brain development was the product of histological or structural analytic approaches in postmortem or clinical samples (Chi et al., 1977; Dobbing & Sands, 1973), or was inferred from RSFC studies conducted in preterm neonates (Doria et al., 2010; Fransson et al., 2007).

Fetal RSFC has enabled us to begin describing properties of typical development as well as the role of the environment in shaping neural network development. Studies of typical development have revealed that network connectivity patterns in utero precede and may guide functional selectivity of certain brain regions, such as the fusiform face area (van den Heuvel et al., 2018), and that macroscale characteristics of fetal functional networks share significant overlap with adult networks (Turk et al., 2019). In addition to shedding light on the origins of typical neural development, fetal RSFC studies also inform our understanding of health risk. For instance, exposures like prenatal stress (Thomason et al., 2021a), cannabis (Thomason et al., 2021b), and lead (Thomason et al., 2019) have been linked to altered fetal neurodevelopment, which has important implications for policy and intervention. Taken together, this work highlights that fetal MRI is a crucial tool for understanding typical and atypical human neurodevelopment and for uncovering the earliest origins of disease risk.

There is need to optimize fetal RSFC analytic pipelines so that this important work can be conducted in a rigorous and reproducible manner. A number of methodological studies have highlighted vulnerabilities in the processing and analysis of RSFC data in adults. For instance, data-driven approaches have revealed that traditional denoising techniques using linear modeling may incorrectly classify intrinsic neural signal as noise (Bright et al., 2017). Furthermore, interlab variation in fMRI processing choices can lead to disparate results, even when labs are analyzing the same data (Botvinik-Nezer et al., 2020). Because of the potent impact of analytic choices on fMRI outcomes, there have been several efforts to create and distribute centralized, robust preprocessing pipelines for adult fMRI data such as fMRI PREP (Esteban et al., 2019) and the Human Connectome Pipeline (Glasser et al., 2013). However, these preprocessing pipelines were not developed to manage the unique challenges inherent to imaging the brain in utero, including high motion, encasement within the maternal compartment, both fetal and maternal sources of noise, and unique geometry of the large field of view and abdominal coil array. There is need for development of fMRI processing pipelines suited to the developmental and methodological considerations specific to the fetus (Rajagopalan et al., 2021).

We elected to focus on three preprocessing steps that require particular attention in the developing brain: normalization to standard space, denoising, and smoothing. One of the largest challenges in fetal fMRI is excessive motion as introduced by both the fetus and by the mother (e.g., breathing). Discarding periods of high motion or excluding subjects whose motion exceeds a stringent threshold is often the first attempt to tackle the problem, but it invariably leads to significant data loss. The balance between maximizing amount of data and maximizing data quality is challenging and highly varied across datasets with different motion profiles. As an additional step, the regression-based motion artifact removal strategy is widely used to control the secondary intravolume effects induced by motion, such as artifacts related to partial voluming and magnetic field inhomogeneities (Friston et al., 1996; Pruim et al., 2015). Typical regression models include 6 to 24 motion covariates derived from the volume realignment (Friston et al., 1996; Yan et al., 2013), yet these covariates are highly reliant on the algorithm used for the realignment and, furthermore, the underlying intravolume effects cannot be captured by the realignment parameters. Beyond motion parameter-based models, spatial independent component analysis (ICA) provides a powerful tool to separate neural-related signal from different sources of noise, including the motion-related artifacts. Applied to fMRI data, ICA decomposes data into a set of spatial independent components and associated time courses (Beckmann & Smith, 2004). Components presenting noise features can subsequently be regressed out of the data. ICA-based denoising is well established as a method for removing motion artifacts in adult imaging, but has yet been evaluated in fetal imaging. Thus, this study examines ICA-based data denoising in a large collection of fetal fMRI scans. Another challenge of the fetal brain is its unparalleled, rapid development across gestation, which complicates the normalization process. For example, it is unclear whether normalizing to a fetal template from a particular stage in gestation (e.g., 32 weeks) is adequate, or if instead it is necessary to normalize to a template that is closely age matched to the fetus (e.g., within a week). Finally, the smaller size of fetal brains compared to adults may require different recommendations regarding smoothing kernel size, which may influence the likelihood of identifying significant associations (Botvinik-Nezer et al., 2020). The present study addresses the effect of these key processing decisions during the preparation of fetal fMRI data for second-level analyses in a large fetal dataset.

## MATERIALS AND METHODS

### Participants

Healthy mothers were recruited during routine obstetrical appointments at Hutzel Women’s Hospital in Detroit, Michigan. Inclusionary criteria included maternal age ≥18 years old, native English speaking, singleton pregnancy, and normal fetal brain anatomy as assessed by ultrasound and MRI examination. MRI visits occurred when fetuses were between 22 and 39 weeks gestational age (GA). This study included data from second- and third-trimester fetuses from a larger ongoing project on fetal brain development who had manually segmented and quality assured raw resting-state fMRI data available at the time of this analysis (*N* = 165). Development of automated processes for fetal brain segmentation is an active area of study (Rutherford et al., 2021), but, at present, manual tracing of the brain is the most precise approach. Additional exclusions were applied for fetuses subsequently born very preterm or with low birth weight (<33 weeks GA, <1,800 g; *n* = 14), those scanned before 25 weeks GA (*n* = 9), and those with fewer than 100 low-motion volumes or high segment-weighted average motion (1.5 mm max excursion, 0.5 mm mean; rotational max >2°, rotation mean >1°, *n* = 21), resulting in a final sample of 121 fetuses (68 male; 53 female). Included fetuses had a mean GA of 32.89 weeks at scanning (*range* = 25.86–39.57; *SD* = 3.75) and were born, on average, at 39.08 (*SD* = 1.49) weeks gestation. More detailed characteristics of the sample are provided in Table 1. Motion parameters were not correlated with demographic variables including scan age and sex in the final sample (see Supporting Information, Figure S1). All study procedures were approved by the Wayne State University Human Investigation Committee.

**Table 1.** Sample demographic characteristics (*N* = 121)

	Mean ± *SD*
Maternal age, years	25.34 ± 4.56
Race/ethnicity, *N* (%)	
Caucasian	10 (8.26%)
African American	99 (81.82%)
Latina	1 (0.83%)
Asian American	1 (0.83%)
Biracial	5 (4.13%)
Not disclosed	5 (4.13%)
Fetal sex, *N* (%)	
Female	53 (43.80%)
Male	68 (56.20%)
Gestational age at scan, weeks	32.89 ± 3.75
Gestational age at birth, weeks	39.08 ± 1.49
Birth weight, grams	3,237.22 ± 510.73

### Data Acquisition

Fetal MRI data were acquired on a Siemens Verio 70-cm open-bore 3T MR system using a 550 g abdominal 4-Channel Siemens Flex Coil (Siemens, Munich, Germany). Twelve minutes of fetal resting-state fMRI data were acquired using the following gradient echo planar imaging sequence: TR/TE 2,000/30 ms, flip angle 80°, 360 frames, axial 4-mm-slice thickness, voxel size 3.4 × 3.4 × 4 mm^3^. The sequence was repeated when time permitted.

### Preprocessing Pipelines

**Figure 1.**
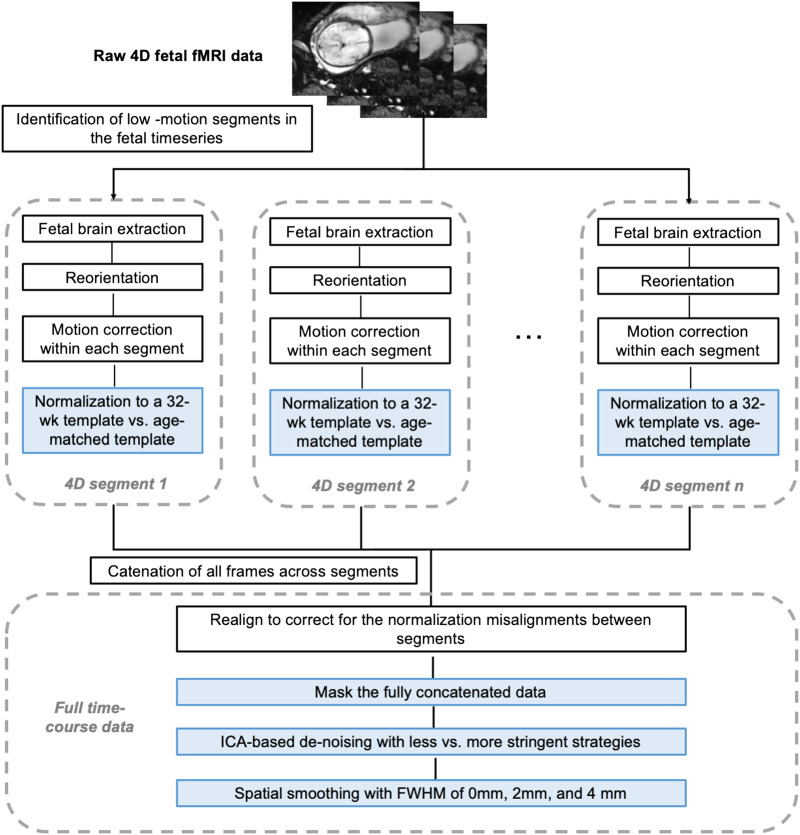
Workflow of the fetal fMRI preprocessing pipeline. Key steps validated in this study are colored by blue boxes.

**Figure 2.**
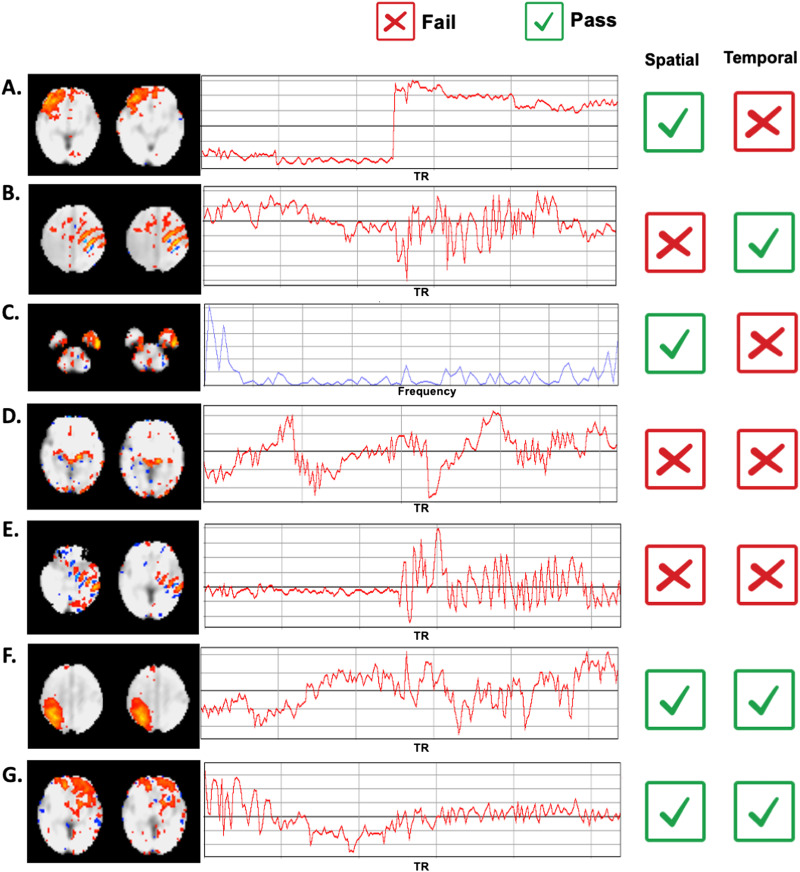
Strategy used for less and more stringent ICA elimination. Exemplar components observed in the fetal dataset are presented above. Observation of a single failure in spatial, temporal, or frequency domains results in elimination of the component, but only at the more stringent level. Less stringent correction only eliminates components if more than one failure is observed, for example, in both spatial and temporal domains. Pass and fail examples are provided here, depicted with checkbox and cross-out, respectively. As examples of single failures, component B shows nonbiological banding patterns (positive/negative stripes), but shows acceptable time course, and component C shows a typical spatial pattern, but shows high-frequency peaks, indicative of scanner-related artifacts. Examples A–C, were eliminated only at the more stringent threshold. D and E show failures in two domains, and F and G pass both spatial and temporal analysis.

A full preprocessing workflow is shown in Figure 1. Time frames in the raw fMRI data corresponding to periods of significant head motion were identified using FSL image viewer (FSL, 2018) and excluded. Brainsuite (Shattuck & Leahy, 2002) was used to manually generate fetal brain masks around single reference images that were then applied to each resulting continuous, low-motion 4D segment. After implementing volume-to-volume motion correction using SPM’s ‘*Realign*’ function within each segment, we then evaluated the effects of different strategies in several key fMRI preprocessing steps:

1. **Normalization to age-matched versus 32-week template**. To assess the influence of different templates on the quality of normalization, we tested normalization from the functional data directly to the standard template of a 32-week GA fetus (mean age for the group) versus to the nearest week-specific template for a given subject (ranging from 25 to 37 weeks GA). Serag’s 2012 templates were used (Serag et al., 2012), and normalization was conducted in SPM using nonlinear warping. The warping metrics were estimated with the first volume of each segment and were then applied to remaining volumes within that segment. Two metrics (Calhoun et al., 2017) were used for comparison: (1) voxel-wise variability of the normalized images across subjects; (2) mean and maximum absolute frame-to-frame displacement derived from performing volume-to-volume realignment, a second time, across the full normalized, concatenated time series. Voxel-wise variability provides a measure for mismatch between fMRI data and the template across subjects. If a given voxel is on the edge and varying constantly between being “in” and “out” of the brain, this voxel will tend to have a high standard deviation. Specifically, the first normalized volume of subjects at the same gestational age were concatenated along the fourth dimension to create a single image file. We calculated the standard deviation of this file along the subject dimension using *Image Calculator* of DPABI toolbox (Yan et al., 2016) implemented in MATLAB. Measurements of absolute displacement of the brain from the original position included total translational movement (maximum and mean difference in position in millimeters) and total head rotation (maximum difference in rotation in degrees; Van Dijk et al., 2012). Scans with more accurate normalization across segments are expected to show lower intersubject displacement (Calhoun et al., 2017). Finally, to explore possible effects resulting from the choice of normalization templates used, we additionally evaluated normalization to alternative age-specific fetal templates (Gholipour et al., 2017). This was a secondary analysis and was thus performed for one representative subject from each gestational age.
  After normalization, segments were concatenated within each scan and potential misalignments between segments were corrected using SPM’s realignment function. For the following processing step comparisons, data resulting from normalization to the 32-week template were used. One subject was excluded here due to low usable frames (*n* = 120 for the following analyses).
2. **Masking the full concatenated data**. To repress background spurious signals, a next step evaluated the utility of applying a dilated brain mask. We tested whether masking at this step improved downstream processing.
3. **Denoising at two stringency thresholds, based on ICA**. Data were decomposed into independent components using FSL’s MELODIC (multivariate exploratory linear optimized decomposition into independent components; Beckmann & Smith, 2004). The number of components was automatically estimated by MELODIC. Noise components were manually labeled twice, once in a less stringent way and once in a more stringent way (Griffanti et al., 2017). With the less stringent method, components showing nonbiological spatial banding patterns, ring-like patterns on edges of the brain, AND high-frequency peaks were labeled as noise; with the more stringent method, components showing banding patterns, ring-like patterns on edges of the brain OR clusters mainly located in the white matter or cerebrospinal fluid OR time series with sudden jumps (caused by segment concatenation) OR significant changes in oscillation patterns OR high-frequency peaks were labeled as noise. In general, the main difference between the two thresholds is whether noise is defined on the basis of temporal or spatial features, alone, or in combination of both. In this study, components showed abnormal temporal features due to segment concatenation, such as sudden jumps (Figure 2A) and alterations of oscillation patterns (Figure 2E), are unique to fetal imaging. Criteria that formed the basis of each exclusion level are depicted in Figure 2. Noise components were removed using the *fsl_regfilt* function. As outlined in the following section, we evaluated the effect of ICA denoising on RSFC measures resulting from each approach.
4. **Smoothing with different kernel sizes**. We tested the effect of smoothing kernels of 0 mm (no smoothing), 2 mm, and 4 mm full-width at half maximum (FWHM) with SPM. The chosen kernels equal 1 or 2 times of our voxel size. We quantified the effects of spatial smoothing by evaluating mean cross-hemisphere functional connectivity strength across different kernel sizes.

### Functional Connectivity Analysis

#### Cross-hemisphere functional connectivity.

We examined RSFC between homotopic voxels in the brain by using the voxel-mirrored homotopic connectivity (VMHC) technique (Zuo et al., 2010), which is a voxel-wise correlation analysis between the images and their left-right mirror version. Preprocessing pipelines were compared on the basis of resultant summary measures of homotopic functional connectivity for each fetus. As an additional means of evaluating the above preprocessing pipeline, global mean VMHC was tested for correlation with the number of frames and head motion parameters derived from the entire time course across subjects using R software (version 4.0.5). Adequate removal of noise in preprocessing steps should be reflected in lack of association of RSFC with frame count and motion.

#### Seed-based functional connectivity.

Seeds were selected to represent regions both distal and proximal to the midline. Seeds were defined manually as spheres with a 3-mm radius (179 voxels), centered on MNI coordinates: (−20.6, 19.8, −8.6), (−7.7, −18.9, −26.6), (−8.6, 13.7, −0.8), (−5.2, −21.4, 8.6), (−5.2, 33.5, −8.6), (−9.5, −5.2, 22.3), (−8.6, −5.2, −4.3), and (−3.4, −39.5, −4.3). These were constructed using Mango Multi-image Analysis software (https://ric.uthscsa.edu/mango/mango.html). Locations approximate the anterior insular, cerebellum, putamen, precuneus, medial prefrontal cortex, supplementary motor area, thalamus, and the visual cortex, respectively, in the 32-week fetal template (Serag et al., 2012). Seeds were selected to approximate locations used in prior research in preterm and term newborns (Smyser et al., 2010) and because functional neural networks related to these seeds are evidenced to be sensitive to early brain development (Thomason et al., 2015). These left hemisphere masks were duplicated for the right hemisphere, resulting in a total of 16 seed regions. Seed regions of interest (ROIs) are represented in Figure S2 of the Supporting Information and files themselves are available online at www.brainnexus.com.

Seed-to-voxel whole-brain analyses were performed on the denoised data in DPABI toolbox. For each subject, the mean time course was extracted from each seed region and correlated with the time course of each voxel throughout the whole brain, yielding individual RSFC maps for each seed region. All RSFC maps were converted to *z*-scores for post hoc analyses. Seed-based RSFC maps of less versus more stringently denoised data were compared using paired two-sample *t* test. Clusters were corrected for whole-brain multiple comparisons by using false discovery rate (FDR) *p* < 0.05. Group mean RSFC maps were estimated by one-sample *t* tests testing the *z*-transformed values against 0, with threshold at *p* < 0.00001 FDR corrected.

#### Group-level ICA as a validation of the proposed pipeline.

In addition to the above voxel-wise and seed-based functional connectivity analyses used for evaluation of preprocessing strategies, we conducted a brain network analysis. To extract group-level intrinsic connectivity networks, we performed spatial ICA implemented in group ICA of Functional MRI Toolbox (GIFT v3.0b, https://trendscenter.org/software/gift/). Optimally preprocessed fMRI data (more stringently denoised, 4-mm FWHM smoothed) were decomposed into 35 spatial components, each of which exhibited a unique time course profile based on the Infomax algorithm. The number of components was estimated based on the image quality by using a minimum description length approach (Rissanen, 1978). A higher order ICA approach was applied to improve functional parcellation (Kiviniemi et al., 2009). Reliability and stability of the algorithm was ensured using ICASSO by repeating the component estimation 20 times (Himberg et al., 2004). Subject-specific spatial maps and time courses were obtained using the back-reconstruction approach (GICA; Calhoun et al., 2004) and converted to *z*-scores.

## RESULTS

### Assessment of Spatial Normalization to Age-Matched Versus 32-Week Template

**Figure 3.**
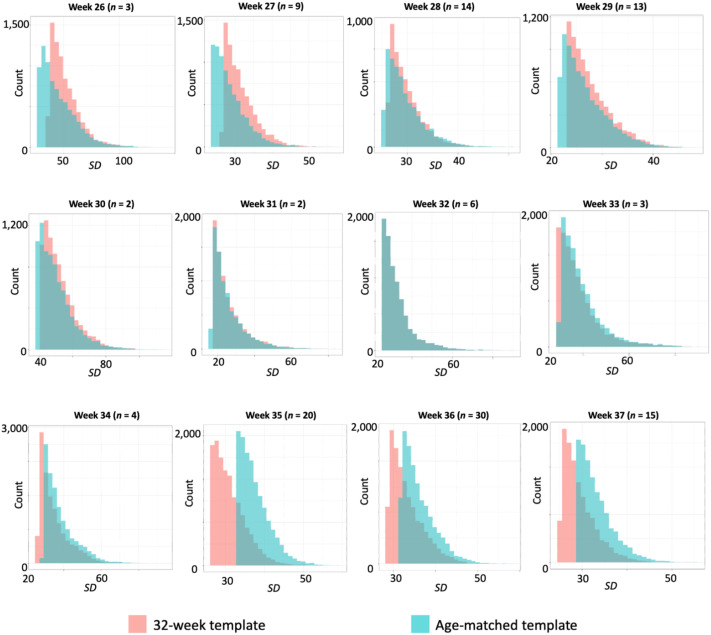
Distributions of standard deviation values after normalization, by fetal age and by template used. Fetuses of different ages were normalized either to a 32-week template (mean for the group) or to a same-age template. Voxels on the edge of the brain have lower standard deviation if they are consistently characterized the same way. The 8,000 voxels with the highest standard deviation are plotted here. Review of observed distributions suggest that the 32-week template performs more optimally for fetuses older than 32 weeks, seen in a leftward shift of 32-week values. The reverse is noted for fetuses younger than 32 weeks, where the age-matched template corresponds to a leftward shift.

When plotting the distribution of 8,000 voxels with the highest standard deviation (Figure 3), we observe that intersubject alignment was improved (*SD* reduced) when using the 32-week template for fetuses older than 32 weeks. However, the reverse was true for younger fetuses; there, an age-matched template was associated with reduced intersubject alignment and better normalization performance. We also observe that the areas of greatest variability are identified at the edges of the brain (see Figure S3 in Supporting Information). This finding may reflect a combination of greater displacement associated with distance from origin and also stronger BOLD signal in cortex compared to white matter and cerebrospinal fluid. We did not find a significant difference in mean and maximum subject-to-subject displacement between the templates at any fetal age (see Figure S4 in Supporting Information). Furthermore, we did not observe a marked difference in normalization performance when an alternate fetal anatomical template set was used (see Figure S5 in Supporting Information).

### Assessment of Individual-Level Masking

**Figure 4.**
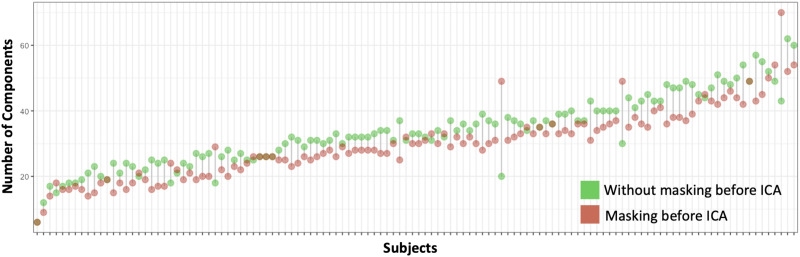
Number of components estimated in FSL’s MELODIC by subjects with and without masking.

With unmasked data, we detected a number of noise components located outside of the brain. We found that masking before denoising reduced the number of ICA-derived components (Figure 4) for most fetal subjects; the total number of independent components across all subjects decreased from 4,043 to 3,623 after reapplying a brain mask. The individual-level masking removed most of the ring-like noise components in the following ICA analysis. This reduction of components alleviates the workload of manual inspection, which is currently needed for fetal data. The dilated mask is shown in Figure S6 of the Supporting Information, and is available online at https://www.brainnexus.com/.

### Assessment of ICA Denoising

The less stringent approach to labeling noise components resulted in the identification of 5% of all ICA-derived components as noise. In contrast, the more stringent approach resulted in 60% of components being labeled as noise. More stringent denoising led to improvement by visual inspection; in particular, we observed reductions in spatial banding patterns, intensity inhomogeneity, and abnormal signal oscillations caused by rapid motion or segment concatenation. Comparison of the two strategies in three representative subjects are shown below for qualitative inspection, following by group-level RSFC comparisons.

**Figure 5.**
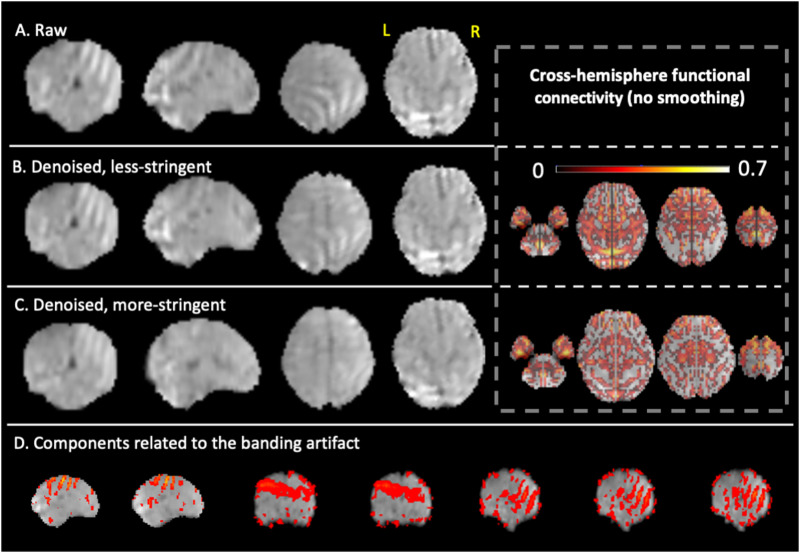
Comparison of less stringent versus more stringent ICA denoising in a representative subject, Case 1. A single volume is shown for a case (35 weeks GA) presenting severe nonbiological banding patterns. (A) Raw data with different planes. (B) Less stringently denoised data with different planes (left), and corresponding cross-hemisphere RSFC (right). (C) More stringently denoised data with different planes (left) and corresponding cross-hemisphere RSFC (right). (D) Examples of ICA noise components related to the banding artifact.

**Figure 6.**
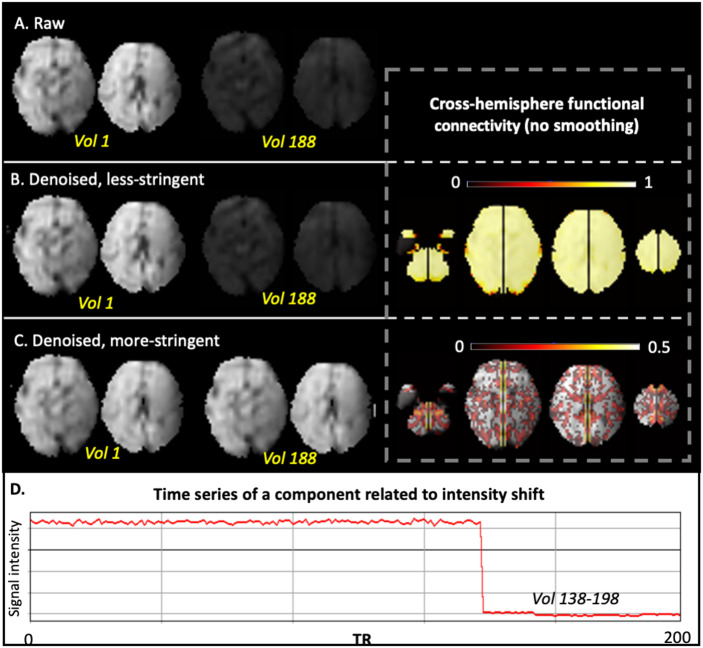
Comparison of less stringent versus more stringent ICA denoising in a representative subject, Case 2. A single volume is shown for a case with noted intensity shift. (A) Raw data of two axial slices at the 1st and 188th volumes. (B) Less stringently denoised data at the 1st and 188th volumes (left) and corresponding cross-hemisphere RSFC (right). (C) More stringently denoised data at the 1st and 188th volumes (left) and corresponding cross-hemisphere RSFC (right). (D) Time series of an example noise component.

**Figure 7.**
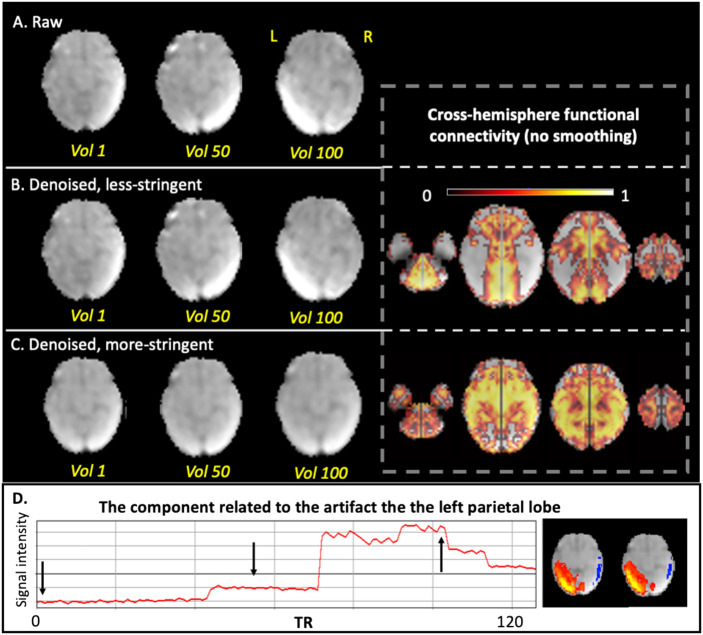
Comparison of less stringent versus more stringent ICA denoising in a representative subject, Case 3. A single volume is shown for a case with high residual motion. (A) Raw data of one slice at the 1st, 50th, and the 100th volumes. (B) Less stringently denoised data at the 1st, 50th, and the 100th volumes (left) and corresponding cross-hemisphere RSFC (right). (C) More stringently denoised data at the 1st, 50th, and the 100th volumes (left) and corresponding cross-hemisphere RSFC (right). (D) The time course and the spatial map of the noise component corresponding to the artifact in left parietal cortex. Arrows indicate the volumes we showed in the above rows.

**Case 1**: A representative subject with severe nonbiological banding patterns in the posterior part of the brain was selected as Case 1. As shown in Figure 5, the nonbiological banding patterns were detected with ICA (bottom row) and were slightly lessened with a less stringent denoising approach. The presence of this nonphysiological banding pattern is usually related to the MRI sequence (e.g., EPI susceptibility or multiband acceleration) or hardware artifacts (e.g., RF interference) or interactions of the acquisition with head motion (e.g., interleaved slice acquisitions) (Griffanti et al., 2017). In contrast, the more stringent denoising further improved homogeneity within the brain and was associated with reduced cross-hemisphere functional connectivity. It is possible that banding patterns remain at the less stringent level of denoising, because even though the spatial pattern is atypical, the time series falls within the normal range.
**Case 2**: When checking components, we noticed an unusual case with massive whole-brain intensity shift during the scan, which was likely due to an issue in the coil or other electronics. With the more stringent strategy, we labeled all components as noise for this subject, as all components shared the same time course that contained a sudden jump. The intensity shift was completely corrected in this approach. As could be expected, without this correction, the cross-hemisphere functional connectivity is biologically implausible across the whole brain (Figure 6).
**Case 3**: In the third subject, there was a sharp increase in intensity in the left parietal cortex in the middle of the scan that corresponded to one ICA-derived component (the bottom row of Figure 7). With more stringent ICA denoising, artifacts were removed (Figure 7) and cross-hemisphere functional connectivity increased. Strong intensity changes in the time course at the joint of two segments, observed in Figure 7D, results from both (1) normalization misalignment between segments and/or (2) fetal repositioning. These signal “jumps” thus reflect inconsistent segment-to-segment spatial alignment (imprecise normalization) or can reflect sizable fetal repositioning with potential to change field geometry or interactions at tissue interfaces.

#### Denoising effects on group-level cross-hemisphere functional connectivity.

**Figure 8.**
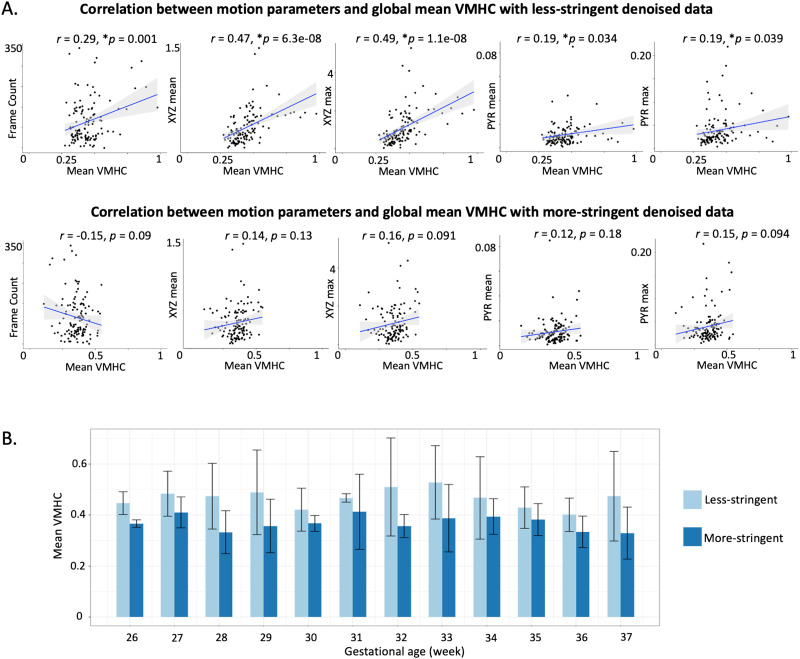
Group-wise comparison of different ICA denoising strategies. (A) Correlations of global mean voxel-mirrored homotopic connectivity (VMHC) with frame counts and motion parameters (XYZ mean and XYZ max for translational movements; PYR mean and PYR max for rotations). (Top row) Less stringently denoised data; (bottom row) more stringently denoised data. (B) Group mean VMHC by age group with less versus more stringent denoising methods. Asterisks (*) in front of *p* values indicate significant correlations.

Global mean cross-hemisphere RSFC was correlated with number of frames (*r* = 0.29, *p* = 0.001) and with motion parameters (mean translational movements: *r* = 0.47, *p* = 6.3e^−08^; maximum translational movements: *r* = 0.49, *p* = 1.1e^−08^; mean rotations: *r* = 0.19, *p* = 0.03; maximum rotations: *r* = 0.19, *p* = 0.039) following less stringent denoising (top row of Figure 8A). After regressing out noise components by using the more stringent approach, the associations between RSFC, frame count, and motion were no longer significant (bottom row of Figure 8A). Overall, a more stringent denoising strategy corresponded to a reduction in cross-hemisphere RSFC (Figure 8B). However, it is notable that decreased cross-hemispheric connectivity was not a ubiquitous feature of denoising; individual cases showed increases in cross-hemispheric connectivity when applying a more stringent ICA. For example, in Case 3 (Figure 7), after removing left-lateralized artifact, we observe increased cross-hemisphere RSFC, suggestive of unmasking underlying connectivity effects.

#### Denoising effects on group-level seed-based functional connectivity.

**Figure 9.**
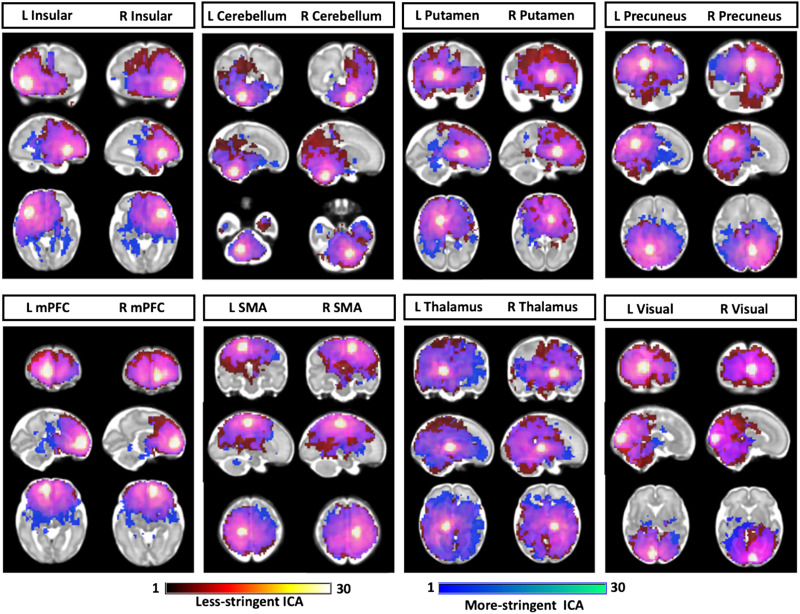
Comparison of seed-based functional connectivity in data analyzed with more or less stringent denoising. One-sample *t* test was sued to compare more stringent ICA (blue) and less stringent ICA (red) (*p* < 0.00001, FDR corrected). Overlapping regions are shown in purple.

Use of different denoising levels was associated with changes in the pattern of RSFC across ROIs, as shown in Figure 9. Select regions showed increased or decreased connectivity at each denoising threshold. Overall, the pattern was such that less stringent denoising was associated with greater overall RSFC, demonstrated in Figure S7 of the Supporting Information.

### Assessment of Spatial Smoothing

**Figure 10.**
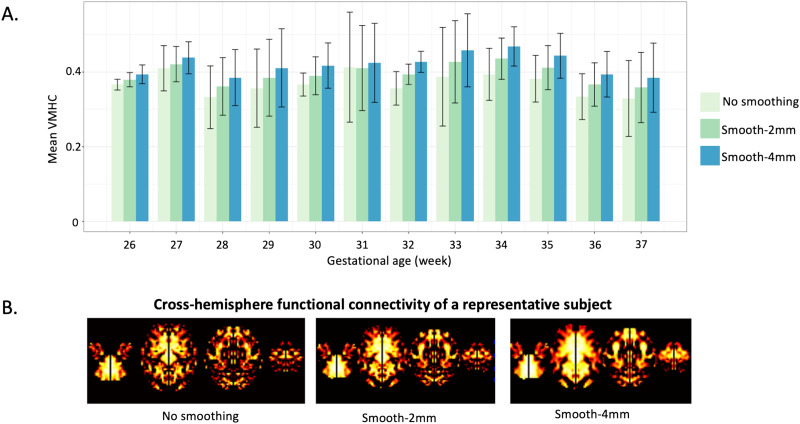
Group-wise comparison of different smoothing kernels. (A) Global mean VMHC changes without or with smoothing kernels of 2 mm and 4 mm. (B) Voxel-wise VMHC of a representative fetus of 37 weeks.

Generally, we observed a dose-dependent relationship between smoothing kernel size and cross-hemisphere functional connectivity, with a 4-mm kernel resulting in the greatest cross-hemisphere functional connectivity. We also note that this effect does not interact with age (Figure 10), suggesting the impact of smoothing kernel size does not vary with fetal age.

### Resting-State Functional Networks in the Fetal Brain

**Figure 11.**
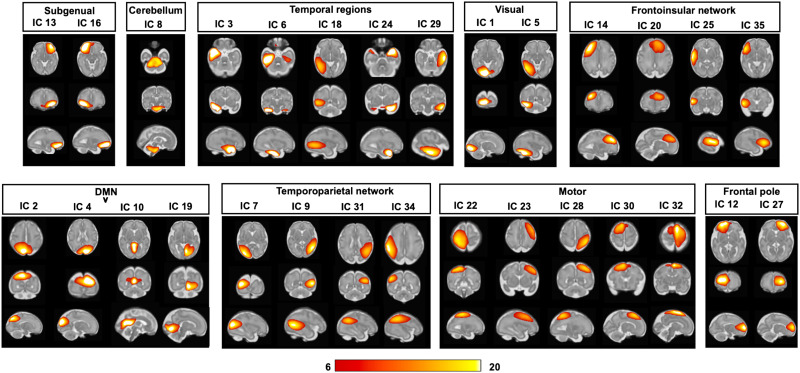
Fetal brain networks derived from ICA. Positive *t* maps threshold at *t* value > 6 are shown.

In an exploratory validation analysis, we examined the presence of fetal resting-state networks in more stringent denoised ICA components (4-mm FWHM smoothed). Thirty-one of the 35 components (available online at www.brainnexus.com) were identified as signal components because their peak coordinates were located primarily in gray matter and their time courses were dominated by low-frequency fluctuations (Allen et al., 2011). We organized the signal components into nine functional networks based on the temporal correlation between the components and the anatomical locations, including the subgenual area, cerebellum, temporal regions, visual network, frontoinsular network, default mode network, temporoparietal network, motor network, and frontal pole areas (Figure 11). Examples of group-level ICA noise are shown in Figure S8 of the Supporting Information. The identified components spatially resembled those previously described in preterm neonates (Smyser et al., 2010) and fetuses (Thomason et al., 2015).

## DISCUSSION

Our analyses confirm that different approaches to normalization, masking, denoising, and smoothing during fetal fMRI preprocessing have notable impacts on data quality. Specific analytic choices during preprocessing impact connectivity metrics derived from BOLD images at the subject level, with implications for reliability and reproducibility of group-level effects.

Results indicate that choice of template relates to normalization variability in an age-dependent manner. Specifically, using a 32-week template resulted in greater normalization accuracy compared to using age-matched templates for fetuses 32 weeks or older. Conversely, using an age-matched template results in greater normalization accuracy for fetuses under 32 weeks gestational age. It is possible that either maturational changes within the brain, variation in size and shape, or some combination of these contribute to this effect. For example, wide difference in size between the source and template images leads to more scaling transformations and increased interpolation. These data also show that when a large fetal age range is studied, fetuses at extreme edges of the range will be most impacted by choice of normalization template. Overall, these observations suggest that template choice may best be determined by research objectives and characteristics of the sample. For example, if age-related development is not the goal of the study, it may be advisable to include gestational age at scan as a nuisance covariate in second-level models and/or to exclude cases to restrict the age range being studied. If the study objective(s) includes age-related development, mean voxel-wise standard deviation can be considered as a regressor to correct for normalization differences across age. In future work it would be valuable to evaluate alternative registration algorithms that are not reliant on off-the-shelf tools.

In ICA-based denoising using FSL’s MELODIC, a high proportion of noise components is usually expected in adults. With standard sequences at 3T, around 70% (Rummel et al., 2013) and 88% (Griffanti et al., 2014) of components may be reported as noise. In our fetal dataset, even using a more stringent strategy, only 60% of components were identified as noise, most of which were motion related. Observing a lower proportion of noise components in fetal fMRI data may be the result of either developmental processes, such as changes in cerebrovascular structure (Reilly & Gutierrez, 2021), or altered noise characteristics unique to this context. As examples of the latter, abdominal versus head coil geometry or field inhomogeneity introduced by large field of view may contribute to differential effects. Another possibility is that a smaller number of components arises simply from larger motion-induced artifacts washing out smaller, more subtle artifacts. Because ICA denoising may perform differently in fetal data, it is advisable that comparisons between fetal and postnatal datasets take this possibility into account.

We also observed that more stringent denoising removed the correlation between all motion parameters and global cross-hemisphere RSFC and resulted in different seed-based RSFC maps. These findings highlight the sensitivity of voxel-to-voxel and seed-to-voxel RSFC to head motion. However, we also want to acknowledge that the more stringent denoising does not guarantee “better” estimates of functional connectivity, considering the risk of potential inadvertent removal of signal. Selection of cross-hemispheric connectivity was the reference analysis used in the present study because it has been evidenced in previous studies a sensitive measure of fetal development (Thomason et al., 2013) and because cerebral homotopy is a fundamental principle of brain organization (Toga & Thompson, 2003). However, it should be noted that this is one of many strategies that could be used to test the effects of denoising on resultant RSFC. This method was useful in confirming that after denoising, an expected pattern in brain organization was observed and was no longer correlated with motion parameters.

In fetal fMRI, motion-related artifact is a significant challenge; motion artifacts can be introduced both by frequent and large-scale changes in fetal position in utero and by maternal respiration (Thomason, 2020). One previous attempt to remove motion-related artifacts in fetal fMRI employed a combined approach of slice to volume registration and scattered data interpolation with bias field and spin history corrections on a small sample of eight fetuses (Ferrazzi et al., 2014). This approach avoids discarding frames, but requires additional scans to assist registration and estimations of field inhomogeneity. In contrast, ICA denoising can be implemented without additional scans. At the single-subject level, ICA-based denoising has proven to be a powerful tool for separating neural-related signal from different sources of noise, including movement artifacts (Griffanti et al., 2017). This is the first study to verify the efficacy of ICA denoising in fetal imaging in a large fetal cohort. Furthermore, the components manually labeled here can be used as a training set for future automatic signal/noise classifiers in fetal imaging data. Given ICA does not guarantee a uniform reconstruction of all frames, especially for high-motion periods, combination of censoring, ICA-based denoising, and covariate regression (e.g., motion, fame count, etc.) may be advisable for mitigating noise in future fetal fMRI studies.

Spatial smoothing can improve signal to noise and reduce the effects of spatial normalization misalignment (Lowe & Sorenson, 1997) at the expense of decreasing resolution. Previous studies on adult brains suggest that the kernel size should be at least twice the size of the fMRI acquisition voxel (Alahmadi, 2021). However, best practices for smoothing parameters in fetal fMRI remain to be addressed. Given the significantly smaller size of fetal brains compared to adult brains, the smoothing kernel recommended in adult imaging may not be appropriate. We observed that using a larger smoothing kernel resulted in enhanced cross-hemisphere functional connectivity in fetuses, fitting with likely improvement in signal to noise. However, given the trade-off between sensitivity and spatial specificity (i.e., the resolution) of findings, general advice in fetal RSFC could be to use a moderate value of ×1 to ×2 voxel size. Best practices in kernel selection will necessarily vary with attributes of the data and with the analytic approach being applied.

While the present study examines key steps in preprocessing fetal fMRI data, it is important to note that many questions remain to be addressed. Fetal data present novel challenges, especially higher and more complex motion and unique image artifacts (van den Heuvel & Thomason, 2016). Methods tested in this article draw from parameters that are most standard in published reports in the literature (Jakab et al., 2014; Thomason et al., 2019; Thomason et al., 2021a; Turk et al., 2019). However, there are alternative emergent approaches, such as that presented by Scheinost and colleagues that perform automatic censoring of low-quality frames and aim to correct for both large and small motion, that are important to explore with advancement of this field of study (Scheinost et al., 2018). It will be valuable for future works to additionally evaluate the efficacy of alternative denoising strategies, such as CompCorr (Behzadi et al., 2007), confound regression and band-pass filtering (Yan et al., 2013), and advanced slice level reconstruction (Ferrazzi et al., 2014), and to do so across datasets with variable noise profiles and motion thresholds. Future studies will also benefit from considering interaction between preprocessing steps, such as whether the choice of normalization target would affect the ICA performance. Additional areas for future work are to test the generalizability of preprocessing steps in data collected from different scanners/vendors, and across in different populations, including clinical samples. Furthermore, it will be useful to evaluate alternative fMRI acquisition techniques, such as multiecho–echo planar imaging (ME-EPI), which lends itself to empirically informed strategies for denoising data during postprocessing (Kundu et al., 2012). Furthermore, an additional acquisition mapping the field may help to address the distortions of the image. Finally, there is great promise in using deep learning algorithms to automate manual steps involved in fetal fMRI data processing, such as brain segmentation (Rutherford et al., 2021). One can imagine further development of these, even for purposes of automated identification of noise and signal components following ICA. Overall, fetal research MRI represents an extraordinary opportunity for basic and clinical science, but it does require continued investment toward optimization and transition to the mainstream. This is elaborated further in a recent commentary (Rajagopalan et al., 2021). The present study addresses common decision points in fMRI data processing and provides empirical comparisons of outputs achieved when applying different methods at each step.

## ACKNOWLEDGMENTS

The authors thank Jasmine Hect and Pavan Jella for their assistance in data acquisition and thank Ava Palopoli and Amyn Majbri for assistance with data management and quality assurance. Importantly, the authors thank participant families who generously shared their time and expressed interest in helping future babies to achieve their best possible health outcomes.

## SUPPORTING INFORMATION

Supporting information for this article is available at https://doi.org/10.1162/netn_a_00254. The data and code used in this study will be made available via https://ndar.nih.gov/ and/or accessed upon direct request to M. E. Thomason (data) or L. Ji (code).

## AUTHOR CONTRIBUTIONS

Lanxin Ji: Conceptualization; Data curation; Formal analysis; Methodology; Software; Visualization; Writing – original draft; Writing – review & editing. Cassandra L. Hendrix: Conceptualization; Visualization; Writing – original draft; Writing – review & editing. Moriah E. Thomason: Conceptualization; Funding acquisition; Resources; Supervision; Writing – review & editing.

## FUNDING INFORMATION

Moriah E. Thomason, Foundation for the National Institutes of Health (https://dx.doi.org/10.13039/100000009), Award ID: MH110793. Moriah E. Thomason, Foundation for the National Institutes of Health (https://dx.doi.org/10.13039/100000009), Award ID: DA050287. Moriah E. Thomason, Foundation for the National Institutes of Health (https://dx.doi.org/10.13039/100000009), Award ID: MH122447. Moriah E. Thomason, Foundation for the National Institutes of Health (https://dx.doi.org/10.13039/100000009), Award ID: ES032294.

## TECHNICAL TERMS

Denoising: A critical step of preprocessing to remove noise and nonneuronal contributions, such as motion-related and physiological noise.
Normalization: A step transforming brains to a template, to ensure that each voxel for each subject corresponds to same brain parts.
Realignment: A step of preprocessing to correct head movements, by co-registering all volumes in a time series to a reference volume.
Independent component analysis (ICA): A data-driven approach to decompose fMRI data into a set of statistically independent spatial maps together with associated time courses.
fMRI preprocessing: A set of image processing steps to clean and standardize fMRI data before statistical analysis.
Spatial smoothing: A step of preprocessing aiming to enhance the signal-to-noise ratio, by averaging data points with their neighbors.
Voxel-mirrored homotopic connectivity (VMHC): A voxel-wise measure of functional connectivity between hemispheres by computing correlations between images and their left-right mirror version.
Seed-based functional connectivity: A voxel-wise correlation analysis between a predefined region, that is, the seed, and all other voxels in the brain.
