# Supplementary material for: Empirical evaluation of human fetal fMRI preprocessing steps
Source: Netw Neurosci. 2022 Jul 1;6(3):702–21. doi: 10.1162/netn_a_00254 (PMC9531599; doi:10.1162/netn_a_00254)
Supplement: Supplementary file 1 [file netn-06-702-s001.pdf]

Ji, L., Hendrix, C. L., Thomason, M. E. (2022). Supporting information for "Empirical evaluation of human fetal fMRI preprocessing steps." *Network Neuroscience*, 6(3), 702–721.
https://doi.org/10.1162/netn_a_00254

Supplementary Materials

Empirical optimization of a human fetal fMRI preprocessing pipeline

Lanxin Ji [1*], Cassandra L. Hendrix [1], Moriah E. Thomason [1,2,3]

[1] Department of Child and Adolescent Psychiatry, New York University Medical Center, New York, NY, USA

[2] Department of Population Health, New York University Medical Center, New York, NY, USA

[3] Neuroscience Institute, New York University Medical Center, New York, NY, USA

Figure S1 Demographic characteristics of included subjects. (A) Distribution of scan age for included subjects. The dashed line shows the sample mean (GA = 32.9 weeks). (B) Correlation matrix of motion parameters with demographic variables, including sex, weight at birth, GA at birth, and GA at scan, in the entire dataset (n = 165). Motion parameters included total translational movement (maximum (XYZ_drift) and mean difference (XYZ_mean) in position in millimeters) and total head rotation (maximum (PYR_drift) and mean difference (PYR_mean) in rotation in degrees). (C) Correlation matrix of motion parameters with demographic variables in the final included sample (n = 121)

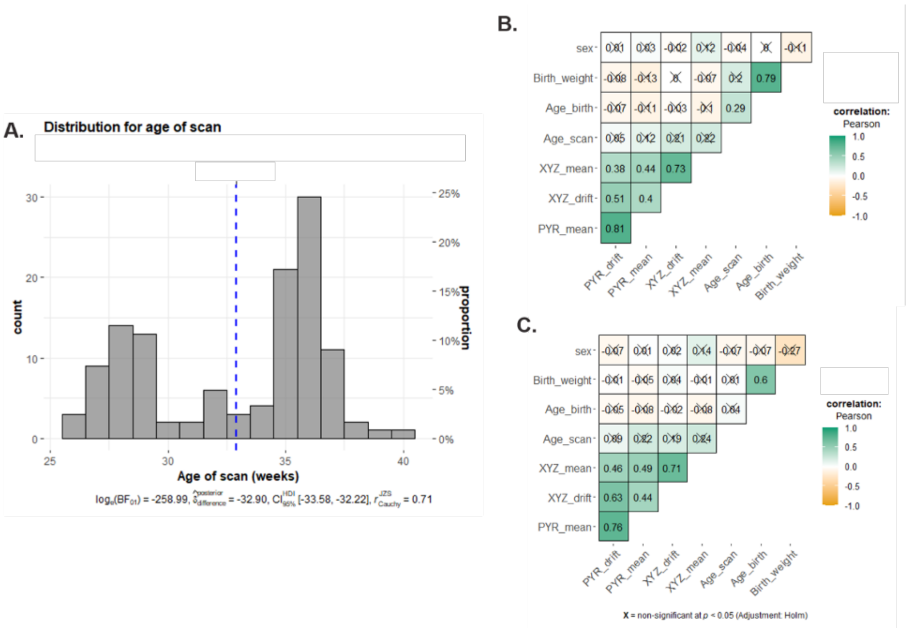

Figure S2. Seeds across GAs, located approximately at the supplementary motor area (SMA), insular, cerebellum, putamen, precuneus, medial prefrontal cortex (mPFC), thalamus, and the visual cortex.

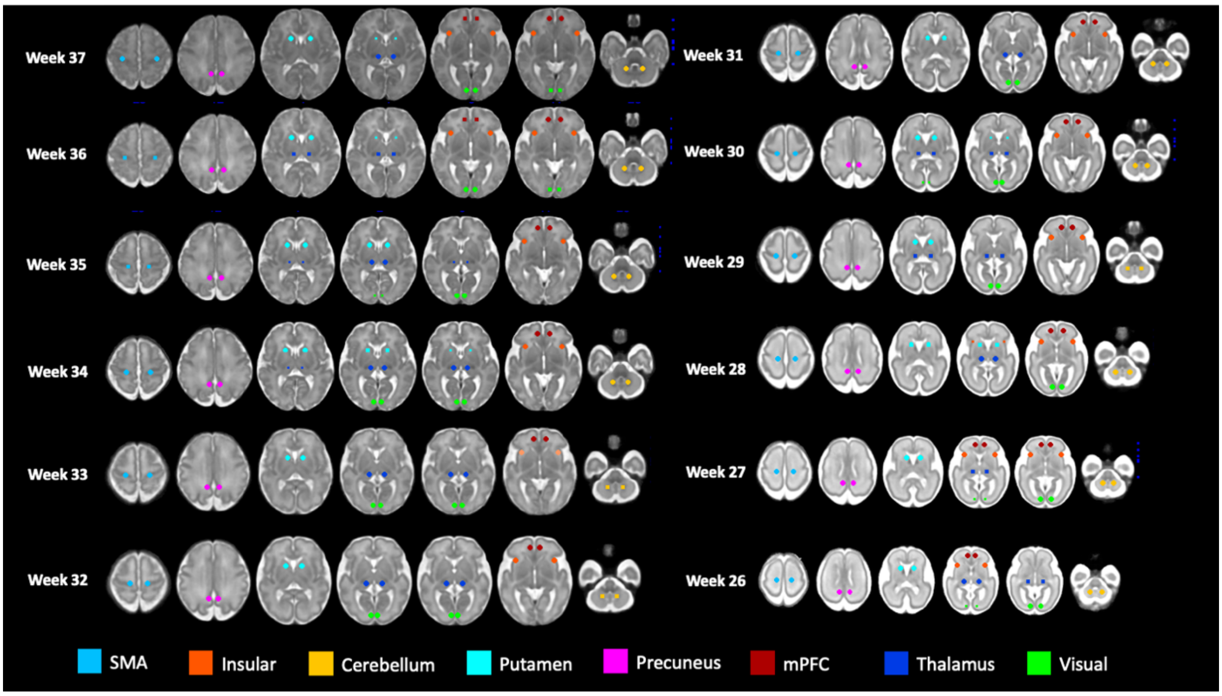

Figure S3. Voxel-wise standard deviation by age group

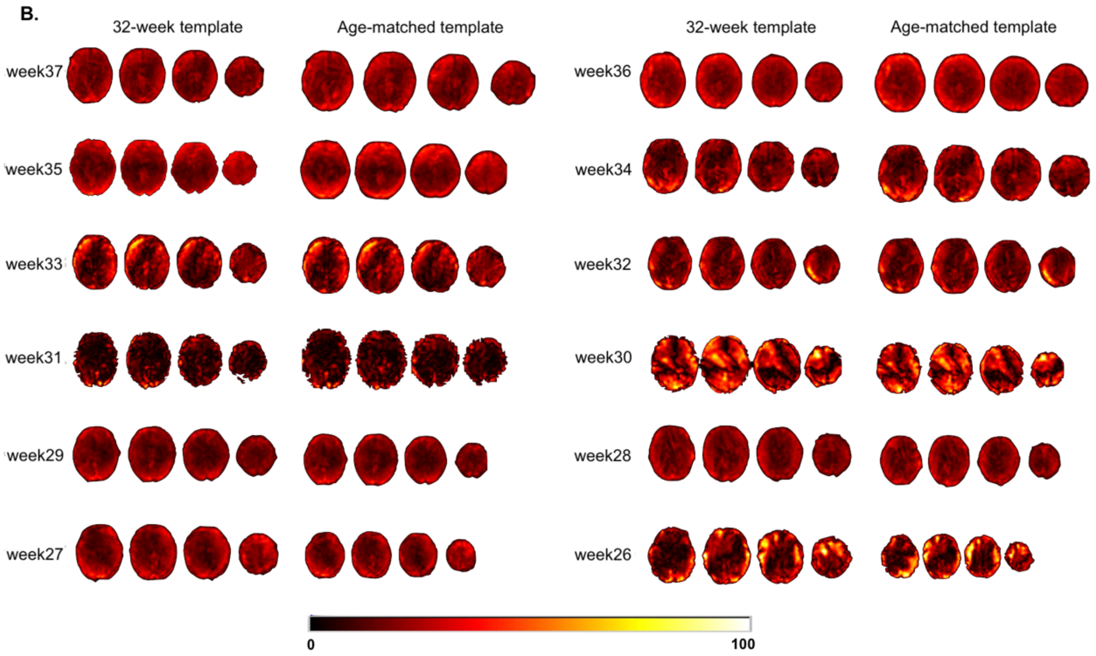

Figure S4. Plots of motion parameters derived from the second realignment by scan age. (top left) Mean translational movement in millimeters. (top right) Mean head rotation in degrees. (bottom left) Maximum translational movement in millimeters. (bottom right) Maximum head rotation in degrees.

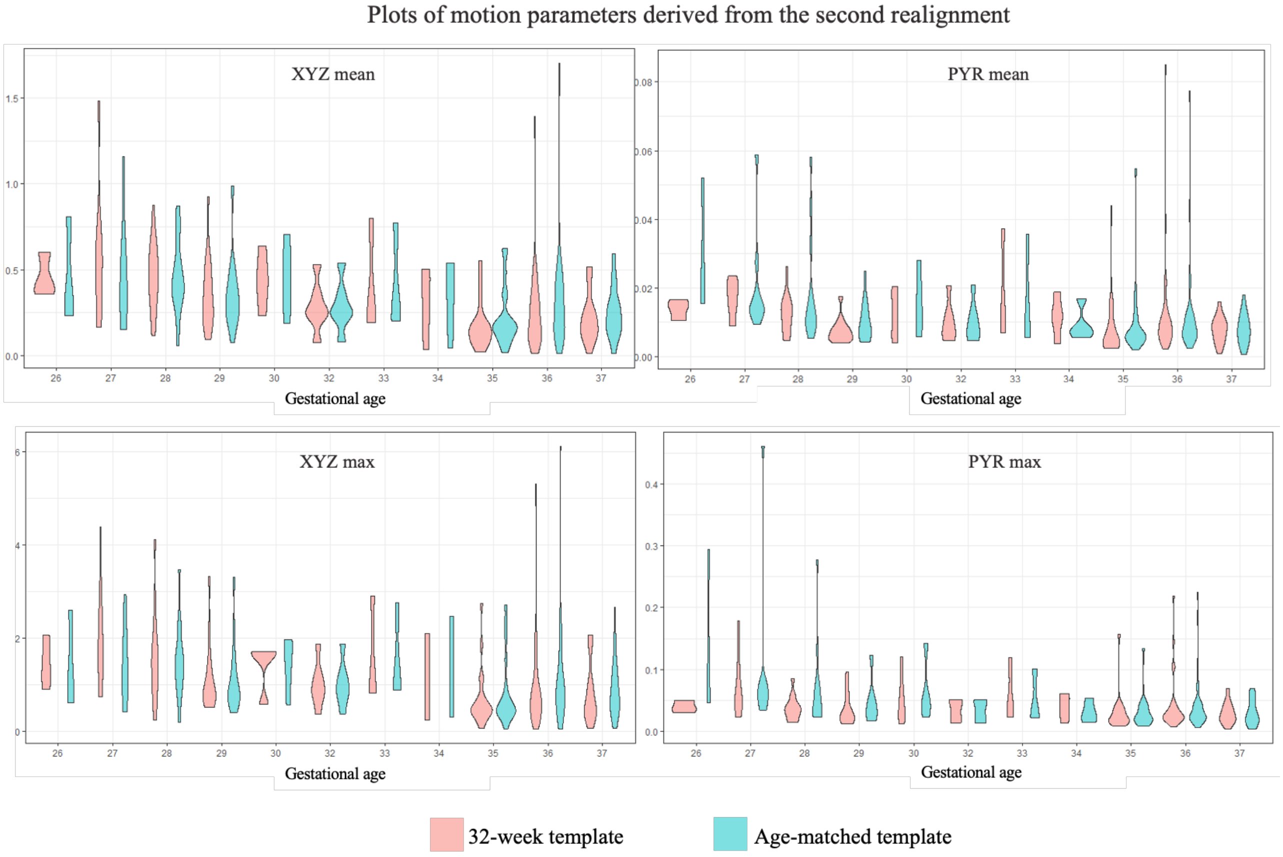

Figure S5. Comparative evaluation of normalization to Gholipour and Serag fetal templates across gestational ages. To explore the effect of the choice of target template, we additionally evaluated normalization to Gholipour templates (Gholipour et al 2017) for one representative subject from each gestational age. We did not observe marked differences in normalization to either template type. In future work it would be valuable to evaluate alternative approaches for comparison of fetal data in representative image spaces that are not reliant on off-the-shelf templates.

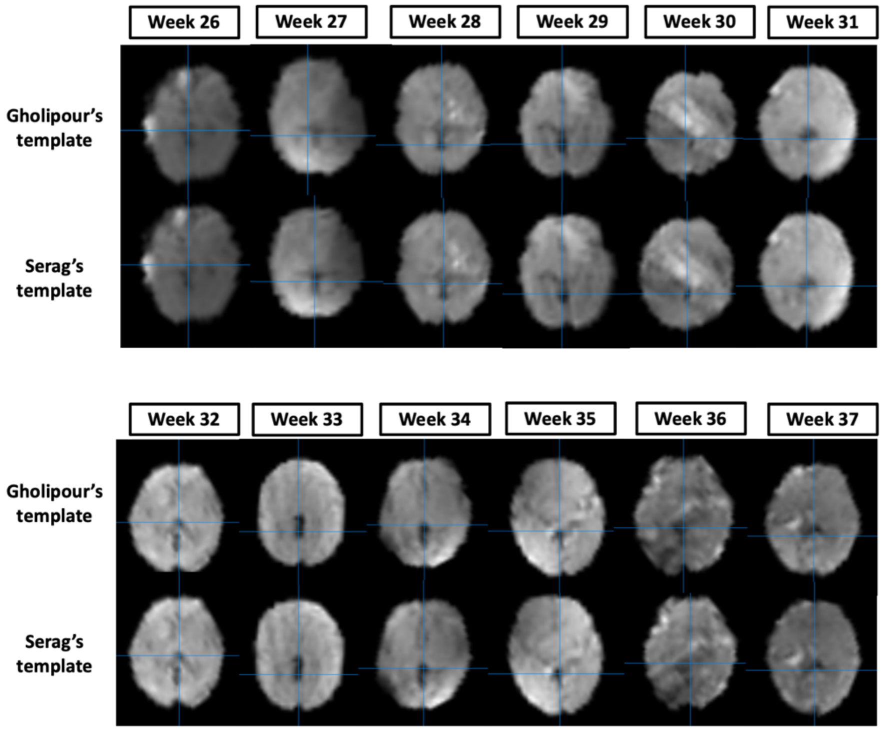

Figure S6. The dilated mask used for individual-level masking before ICA-denoising.

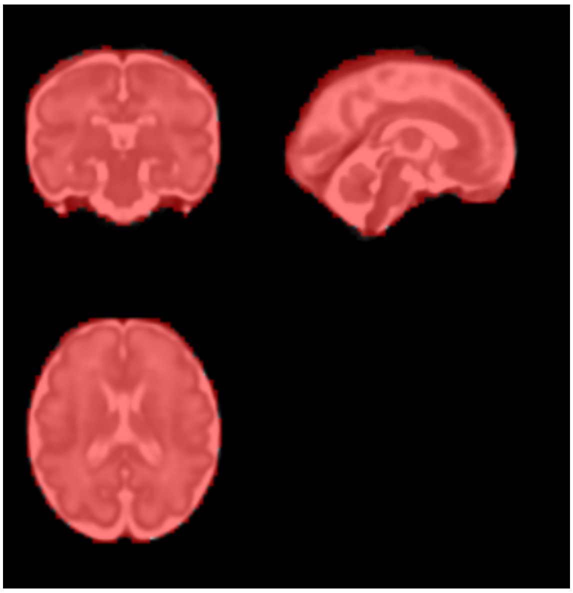

Figure S7. Paired t-test on seed-based functional connectivity between less-stringently and more-stringently denoised data ($p < 0.05$, FDR corrected). Seeds are labeled in red. Following more-stringent ICA denoising, significantly lower RSFC was identified with all seeds except for the bilateral thalamus.

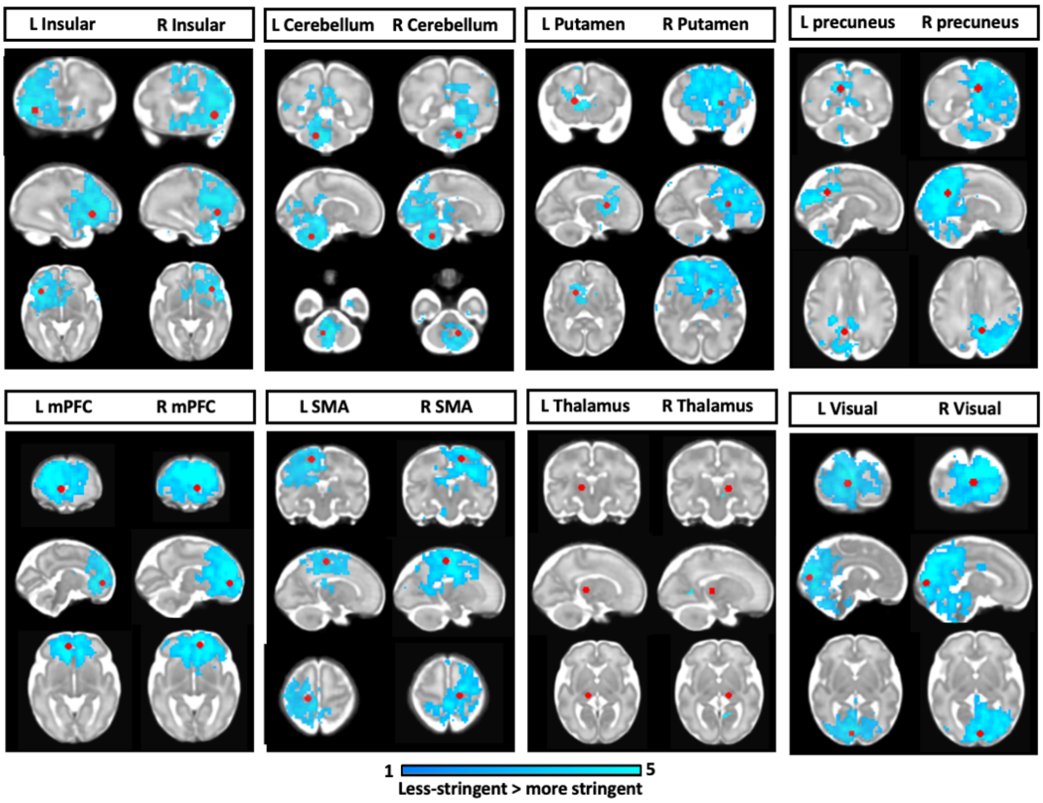

Figure S8. Exampled group-level noise components.

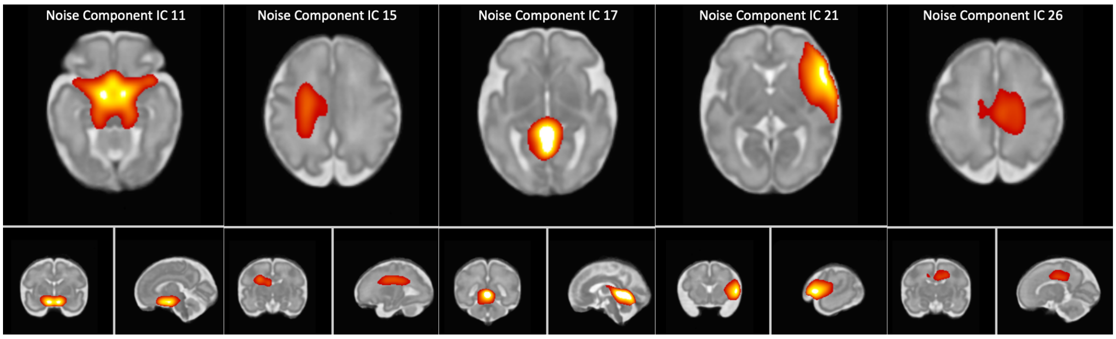

Reference

Gholipour A, Rollins CK, Velasco-Annis C, Ouaalam A, Akhondi-Asl A, et al. 2017. A normative spatiotemporal MRI atlas of the fetal brain for automatic segmentation and analysis of early brain growth. *Scientific reports* 7: 1-13
